# Clinical Characteristics, Diagnosis, Treatment, and Mortality Rate of TB/COVID-19 Coinfectetd Patients: A Systematic Review

**DOI:** 10.3389/fmed.2021.740593

**Published:** 2021-12-01

**Authors:** Maryam Koupaei, Adel Naimi, Narges Moafi, Paria Mohammadi, Faezeh Sadat Tabatabaei, Soroosh Ghazizadeh, Mohsen Heidary, Saeed Khoshnood

**Affiliations:** ^1^Department of Microbiology and Immunology, School of Medicine, Kashan University of Medical Sciences, Kashan, Iran; ^2^Cellular and Molecular Research Center, Sabzevar University of Medical Sciences, Sabzevar, Iran; ^3^Department of Laboratory Sciences, School of Paramedical Sciences, Sabzevar University of Medical Sciences, Sabzevar, Iran; ^4^Clinical Microbiology Research Center, Ilam University of Medical Sciences, Ilam, Iran

**Keywords:** tuberculosis, COVID-19, review, coinfection, TB

## Abstract

**Introduction:** Novel coronavirus (COVID-19) and tuberculosis (TB) are the newest and one of the oldest global threats, respectively. In the COVID-19 era, due to the health system's focus on the COVID-19 epidemic, the national TB control program received less attention, leading to a worsening of the global TB epidemic. In this study, we will review the characteristics of TB patients coinfected with COVID-19.

**Material and Methods:** Using Scopus, PubMed/Medline, Embase, and Web of Science databases, a systematic search was performed. Case reports and case series on TB/COVID-19 coinfection published from January 1, 2019 to February 24, 2021 were collected. There were no limitations regarding publication language.

**Results:** Eleven case series and 20 case reports were identified from 18 countries, with the majority them being from India (*N* = 6) and China (*N* = 4). Overall, 146 patients (114 men and 32 women) coinfected with TB and COVID-19 enrolled. Smoking (15.1%), diabetes (14.4%), and hypertension (8.9%) were the most frequent comorbidities among these patients. The COVID-19 patients with TB mainly suffered fever (78.8%), cough (63.7%), and respiratory distress (22.6%). Hydroxychloroquine (64.0%) and lopinavir/ritonavir (39.5%) were the most common treatments for them. The mortality rate was 13.0% and the rate of discharged patients was 87.0%.

**Conclusion:** Global prevalence of COVID-19-related deaths is 6.6%. Our results showed that 13.0% of patients with TB/COVID-19 died. Thus, this study indicated that coinfection of TB and COVID-19 can increase the mortality. The respiratory symptoms of TB and COVID-19 are very similar, and this causes them to be misdiagnosed. In addition, TB is sometimes diagnosed later than COVID-19 and the severity of the disease worsens, especially in patients with underlying conditions. Therefore, patients with TB should be screened regularly in the COVID-19 era to prevent the spread of the TB/COVID-19 coinfection.

## Introduction

The most recent problem in the world today is the spread of the 2019 novel coronavirus (2019-nCoV). The virus originated in China and is now affecting many countries around the world (1). COVID-19 is the name of the respiratory syndrome caused by 2019-nCoV (2). Various symptoms, including fever, breathlessness, and lung lesions are seen in infected persons (3). Interhuman transmission occurs in hospitals and medical staff and also among family members (4). *Mycobacterium tuberculosis* (*M. tuberculosis*) is the causative agent of tuberculosis (TB) (5, 6). Airborne transmission is the most common method in which *M. tuberculosis* spreads between healthy and TB-active infected persons (7). The prevalence of TB in 2019 is approximately 10 million people and decreases by only 1–2% every year (8). Contact with people coinfected with TB and HIV is one of the main risk factors to increase the prevalence of TB (9). Social factors such as poverty, living environment, population, and economic status affect the COVID-19 and TB incidence. Therefore, improving social life can be effective in controlling TB and COVID-19 (10). Bacterial coinfection is a common problem in COVID-19 patients (11). During the outbreak of COVID-19, many cases of TB/COVID-19 coinfection were reported (12–16). Some studies reported death in coinfected patients with TB and COVID-19 (16–18). A previous study has shown an association between the high incidence of COVID-19 in TB patients (19). Both COVID-19 and TB have respiratory symptoms (20). The presence of respiratory diseases in patients causes dysfunction of the respiratory system and lungs, making TB a risk factor for COVID-19 (19). Prioritizing financial and human resources to combat the COVID-19 epidemic and increasing the risk of SARS-CoV-2 in people with TB raises concerns about TB control. These may reverse the achievements of TB control and increase the prevalence of TB in the future (10, 21). In this systematic review, we reviewed the case reports and case series presenting the TB/COVID-19 coinfection to evaluate the various aspects such as symptoms, diagnosis, treatment, and mortality rate.

## Materials and Methods

This systematic review was performed according to preferred reporting items for systematic reviews and meta-analyses (PRISMA) statement (22).

### Information Source and Search Strategies

We performed a systematic review using Web of Science, Embase, Scopus, and PubMed/Medline. Search criteria included case series and case reports articles published from January 1, 2019 to February 24, 2021. There were no limitations regarding the publication language, but for eligible non-English studies, we used Google Translate. The search terms for our review included *Mycobacterium tuberculosis*, tuberculosis, COVID-19, severe acute respiratory syndrome coronavirus 2, novel coronavirus, SARS-CoV-2, nCoV disease, SARS2, 2019-nCoV disease, coronavirus disease-19, coronavirus disease 2019, novel coronavirus 2019, Wuhan coronavirus, Wuhan seafood market pneumonia virus, and Wuhan pneumonia outbreak.

### Study Selection

Studies included in this review met the following criteria: retrospective, descriptive, and prospective case series and case reports of COVID-19 in hospital settings. The studies presenting diagnostic methods, laboratory and clinical features, and treatment and its outcomes were also included. Reviews, publications without peer review processes, and papers describing experimental approaches were excluded. All potentially related articles followed two steps for eligibility. In the first step, two independent reviewers screened the titles and abstracts of the articles and eliminated duplicate papers using the EndNote X7 program (Thomson Reuters, New York, NY, USA). In the second step of evaluation, the same reviewers retrieved and reviewed the full text of those abstracts that met the inclusion criteria. Technical uncertainties and disagreements were resolved between the authors.

### Data Extraction

The extracted data entailed first author's name, study country, year of publication, type of the study, number of co-infected patients, median age, gender, TB diagnostic method, TB treatment, TB disease, SARS-CoV-2 diagnosis method, clinical manifestations, comorbidities, and outcomes. Three authors independently extracted the data from the selected articles. The reviewed data was jointly reconciled, and disagreements resolved between authors.

### Quality Assessment

The critical appraisal checklist for case reports and case series provided by the Joanna Briggs Institute (JBI) was used for the qualitative evaluation of the studies (23).

## Results

**Figure 1:**
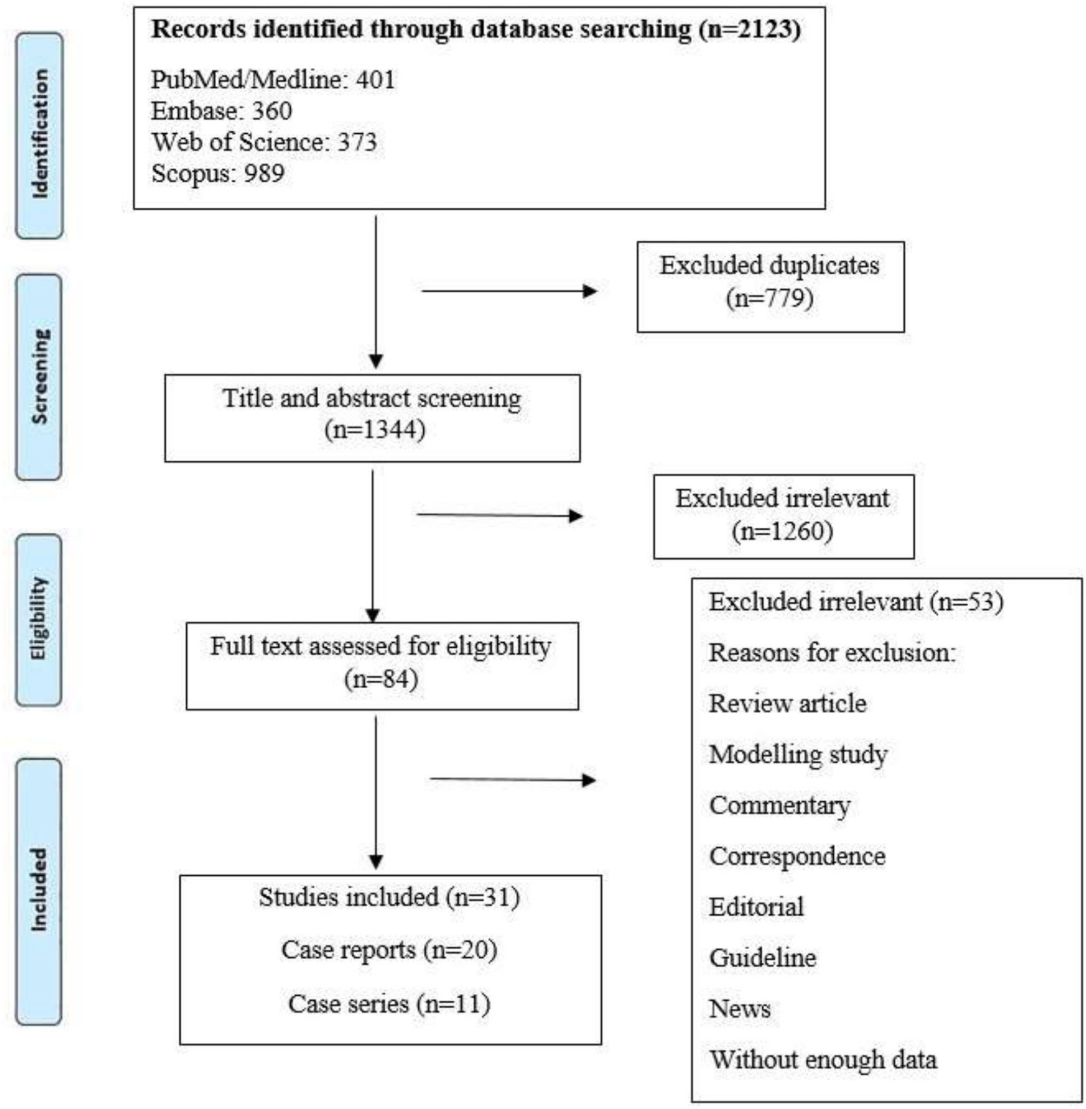
Flow diagram detailing review process and study selection.

Our systematic search resulted in 2,123 potentially relevant articles, of which 1,260 were excluded after title and abstract evaluation (Figure 1). By applying the inclusion/exclusion criteria to the full-text documents, we found that 31 articles were eligible for inclusion in our systematic review. A total of 146 TB/COVID-19 coinfected patients were confirmed with different methods.

In the search, we detected 11 case series and 20 case reports from 18 countries, and the majority of studies were from India (*N* = 6) and China (*N* = 4). Characteristics of the eligible studies are listed in Table 1. We included case reports in our review because they reported new laboratory findings, new CT findings and clinical features, atypical manifestations, treatment outcomes, and some of these reports were the first in a specific country.

**Table 1:** Characteristics of the included studies.

**References**	**Country**	**Published time**	**Type of study**	**Number of co-infected patients**	**Median** **age**	**Male/** **female**	**TB diagnosis method**	**TB treatment**	**TB** **disease**	**SARS-CoV-2 diagnosis method**	**COVID-19 treatment**	**Clinical manifesta-** **tions**	**Other comorbi-** **dities**	**Outcomes**
Tham et al. (24)	Singapore	November 2020	Case series	4	31.75	4M	CXR, Smear Culture IGRA	4 RIF, 4 INH, 4 PYR,4 ETB	4 Pulmonary	CT-ScanRT-PCR	NM	4 Fever, 4 Cough, 1 Dyspnea, 1 Chest pain, 1 Weight loss, 4 Ground glass opacities	None	4 Discharge
Yao et al. (16)	China	November2020	Case series	3	50.3	3M	Smear CXR	2 RIF, 2 INH, 2 PYR,2 ETB	3 Pulmonary	CT-ScanRT-PCR	2 Lop/Rit, 1 UMF HCL, 2 IFN-α, 2 Corticosteroid, 1 IG, 2 Oxygen therapy	3 Fever, 3 Cough, 3 Fatigue, 3 Wheeze, 1 Chills, 3 Weight loss, 1 Night sweats, 1 Tachypnea, 1 Tachycardia, 3 Ground glass opacities	2 Smoking, 1 Diabetes	1 Death, 2 Discharge
Liu et al. (25)	China	May 2020	Case series	3	40	3M	CXR, Smear Culture IGRA	1 ETB, 1 PYR, 1 AMK, 1 CS, 1 LEV, 1 CFZ, 1 LNZ	3 Pulmonary	CT-ScanRT-PCR	1 DEX, 1 AZT, 3 Arbidol 3 MOX, 2 LNZ, thymosin, 1 Oxygen therapy	3 Fever, 1 Fatigue,2 Dyspnea, 2 Cough, 1 Myalgia, 1 Sore throat, 1 Chest pain, 1 Emphysema,1 anxious, 2 respiratory distress, 2 hypoxia, 1 sepsis, 2 hemoptysis, 3 Ground glass opacities	1 Low cellular immunity, 1 Aspergillus fumigatus infection, 1 MDR-TB	3 Discharge
Gupta et al. (26)	India	November, 2020	Case series	22	40.6	20M/2F	NM	22 RIF, 22 INH, 22 PYR,22 ETB, 1 MDR-therapy	17 Pulmonary, 4 Extra-pulmonary (1 CNS, 1 Cervical, 2 pleural), 1 Disseminated	CT-Scan	NM	18 Fever, 1 headache,11 cough, 7 Respiratory distress, 1 Weight loss	3 Diabetes, 4 hypertension, 2 seizure disorder, 1 MDR-TB	6 Death, 16 Discharge
Lopinto et al. (27)	France	September 2020	Case report	1	58	M	CXR	NM	Pulmonary	CT-Scan	Oxygen therapy	Fever, cough, increased expectoration, respiratory distress, myalgia, asthenia, hemoptysis, dyspnea. Ground glass opacities	None	Discharge
Pinheiro et al. (13)	Brazil	November, 2020	Case report	1	68	M	GeneXpert	NM	Pulmonary	Solorological rapid test, CT-Scan	NM	Dyspnea, cough, fever, Ground glass opacities	Diabetes, hypertension, chronic liver disease, schistosomiasis	NM
Goussard et al. (28)	South africa	March2020	Case report	1	2.5	M	GeneXpert, CXR	RIF, INH, PYR,ETB	Pulmonary	RT-PCR,	Oxygen therapy, Co-AMX, Prednisone	Fever, cough, respiratory distress, poor appetite	Without BCG vaccination	Discharge
Orozco et al. (29)	Mexico	November2020	Case report	1	51	1M	Smear, GeneXpert	RIF, INH, PYR,ETB	Pulmonary	RT-PCR, CT-Scan	Oxygen therapy	anosmia, dysgeusia, nocturnal diaphoresis, cough, respiratory distress, Ground glass opacities	Diabetes	Discharge
Rivas et al. (30)	Panama	August 2020	Case series	2	41	2M	GeneXpert, CXR	2 RIF, 2 INH, 2 PYR,2 ETB	Pulmonary	RT-PCR,CT-Scan	2 AZT, 2 HCQ, 2 Oxygen therapy, 1 CTX, 1 LEV, 1 Tenofovir, 1 Lamivudine, 1 Dolutegravi	2 Fever, 1 Cough, 2 Dyspnea, 1 weigh lose, 1 Tachypnea	2 HIV, 2 Anemia	2 Discharge
Musso et al. (31)	Italia	January2020	Case report	1	45	M	IGRA PCR, Smear, CXR	RIF, INH, ETB, PYR, AMK, MOX	Pulmonary	Clinical features	HCQ, Corticosteroids	Cough, fever, fatigue, weight loss, Tachycardia, Emphysema, Ground glass opacities	Immune-suppression	Death
Ata et al. (12)	India	August,2020	Case report	1	28	M	GeneXpert, CXR, IGRA	RIF, INH, PYR,ETB, Pyridoxine	Pulmonary	RT-PCR	AZT, HCQ	Dizziness, headache, vomiting, Ground glass opacities	Glioma	Discharge
Faqihi et al. (32)	Saudi Arabia	July,2020	Case report	1	60	M	GeneXpert	RIF, INH, PYR,ETB, Pyridoxine	Pulmonary	RT-PCR, CT-Scan	LOP/Rit, RIB, DEX, ACs, Oxygen therapy	Fever, cough, chest pain, myalgias, fatigue, respiratory distress, Ground glass opacities	Diabetes, hypertension	Discharge
Freij et al. (18)	America	September,2020	Case report	1	5	F	PCR, Culture	None	Extra-pulmonary (CNS)	RT-PCR	AMX, HCQ,AZT	Fever, headache, Brain inflammation	Group A streptococcal pharyngitis	Death
Bouare et al. (33)	Africa	July,2020	Case report	1	32	F	PCR, Smear, CXR	INH, RIF, PYR, ETB	Pulmonary	RT-PCR, CT-Scan	HCQ, AZT, Oxygen therapy	Respiratory distress, fever, cough, headache, myalgia	HIV, Anemia	Discharge
Farias et al. (34)	Brazil	August, 2020	Case series	2	41	2M	Smear, GeneXpert	2 RIF, 2 INH, 2 PYR,2 ETB	Pulmonary	RT-PCR, CT-Scan	2 HCQ, 2 AZT, 2 CTX, CS, 2 Oxygen therapy	1 Fever, 1 myalgia, 1 headache, 1 cough, 2 respiratory distress, 2 glass-ground opacities,	1 HIV, 1 Hepatitis B, 1 Drug abuse	2 Discharge
Vilbrun et al. (35)	Haiti	September 2020	Case report	1	26	M	Smear, Culture, CXR, GeneXpert	BDQ, LEV, LNZ, CFZ PYR,FQ, CS	Pulmonary	RT-PCR, CT-Scan	NM	Cough, fever, weight loss	MDR-TB	Discharge
AlKhateeb et al. (36)	South Asian	November2020	Case report	1	28	M	Smear, Culture, IGRA, CXR, PCR	RIF, INH, PYR,ETB,pyridoxine	Extra-pulmonary (PNS)	RT-PCR, CT-Scan	NM	Cough, fever, night sweats, weight loss, Respiratory distress, chills	None	Discharge
Çinar et al. (37)	Turkey	May 2020	Case report	1	55	M	NM	RIF, INH, PYR,ETB	Disseminated	CT-Scan	Plasma therapy, FAV, TCZ	Fever, Cough, tachycardia, ground-glass opacities	MDS/RAEB myeloid neoplastic disease, Kidney disease, Klebsiella pneumoniae infection	Discharge
Yadav et al. (15)	India	August 2020	Case report	1	43	M	Smear, CXR, GeneXpert	RIF, INH, PYR,ETB	Pulmonary	RT-PCR	Oxygen therapy	Cough, chestpain, poor appetite, fever, chills, night sweats, Respiratory distress, dyspnea	None	Discharge
Gersteina et al. (38)	El Salvador	January 2021	Case report	1	49	M	Smear, Culture, CXR, IGRA	RIF, INH, ETB, LEVPYR	Extra-pulmonary (Abdominal)	RT-PCR	Plasma therapy, HCQ, Oxygen therapy	Fever, Cough, Respiratory distress, Abdominal pain, abdominal distension, orthopnea	Burning, Alcoholism, cirrhosis, hypertension	Discharge
Gajbhiye et al. (39)	India	April 2021	Case series	6	23.5	6F	Smear, Culture, CXR,	NM	6 Pulmonary	RT-PCR, CT-Scan	1 HCQ, 1 Corticosteroids	2 Fever, 3 cough, 2 Respiratory distress	6 Pregnancy, 1 MDR-TB, 1 XDR-TB, 2 ARDS, 1 Preeclampsia	1 Death, 5 Discharge
Baskaraet al. (40)	Indonesia	January 2021		1	42	M	GeneXpert	INH, RIF, PYRETB	Pulmonary	Serology, CT-Scan	OSE, HCQ, ACs	Dizziness, Headache, shivering, cough, abdominal pain, night sweats, ground-glass opacities	Diabetes	Discharge
Culter et al. (41)	USA	September2020	Case report	1	61	M	Smear, CXR, GeneXpert	INH, RIF, PYRETB,	Pulmonary	RT-PCR, CT-Scan	HCQ, Oxygen therapy	Cough, fever	Parkinson's	Discharge
Cao et al. (14)	China	July2020	Case report	1	47	F	CXR	RIF, INH, PYR,ETB	Pulmonary	RT-PCR,CT-Scan	Lop/Rit	Fair mental status, poor sleep, poor appetite	Asthma, hemoptysis	Discharge
Gbenga et al. (42)	Nigeria	October2020	Case series	2	31.5	2M	GeneXpert, CXR	2 RIF, 2 INH,2 PYR, 2 ETB	Pulmonary	RT-PCR	AZT, Vit C, Zinc Sulfate, Prednisone, Lop/Rit	2 Cough, 2 Fever, 2 weight loss, 1 Respiratory distress, 1 Sore throat, 1 Tachypnea	None	2 Discharge
Luciani et al. (43)	Switzerland	October 2020	Case report	1	32	F	CXR, GeneXpert	INH,RIF, PYR,ETB	Pulmonary	RT-PCR	AMX/CA, Paracetamol, Lop/Rit, HCQ, Clarithromycin, Piperacillin/tazobactam	Fever, muscle pain	None	Discharge
Khayat et al. (44)	Saudi Arabia	January2021	Case report	1	40	F	CXR, culture, PCR	RIF, INH, PYR, ETB	Pulmonary	CT-Scan,RT-PCR	NM	Fever, cough, body aches, chest pain,anorexia	None	Discharge
He et al. (45)	China	January2020	Case series	3	56.3	3M	CXR, culture, smear, GeneXpert	1 RIF,1 INH,1 PYR, 1 ETB	3 Pulmonary	RT-PCR,CT-Scan	3 Lop/Rit, 3 arbidol, 2 MP, 2 Oxygen therapy	3 Cough, 3 Fever, 1 chest pain, 1 diarrhea, 2 dyspnea, 2 Chest tightness, 3 Ground glass opacities	2 Hyoxemia, 3 Bacterial infection, 3 ARDS	3 Discharge
Singh et al. (46)	India	August 2020	Case report	1	76	F	CXR, culture, smear, GeneXpert	RIF, INH, PYR, ETB	Pulmonary	RT-PCR	AZT, MP, HCQ, Vit C, acetyl cysteine	Fever, Respiratory distress, Cough, Poor appetite, Weight loss	Hypertension	Discharge
Tadolini et al. (17)	Multi-center	April2021	Case series	49	48	40M/9F	NM	NM	36 Pulmonary, 1 Extra-pulmonary, 12 Both	RT-PCR, CT-Scan	41 HCQ, 25 Lop/Rit, 13 AZT, 2 acetyl cysteine	32 Fever, 27 Cough, 17 Dyspnea, 21 Ground glass opacities	8 COPD/ asthma, 8 Diabetes, 6 HIV, 5 Kidney disease, 7 Liver disease, 10 Alcoholism, 20 Smoking, 4 Drug abuse	5 Death, 44 Discharge
Sarinoglu et al. (47)	Turkey	July2020	Case series	30	38.3	21M/9F	Culture, smear, GeneXpert	NM	NM	RT-PCR	NM	30 Fever, 20 Cough, 10 Respiratory distress	5 Hypertension, 6 Heart disease, 9 Lung disease, 5 Diabetes, 8 Immunosuppression	NM

*NM, Not mentioned; LEV, Levofloxacin; AZT, Azithromycin; HCQ, Hydroxychloroquine; CXR, Chest x-ray; IG, Immunoglobulin; CTX, Ceftriaxone; IGRAs, Interferon-Gamma Release Assays; Co-AMX, Co_amoxiclav; QFT-Plus, QuantiFERON TB Plus; AMK, Amikacin; Lop/Rit, Lopinavir/ritonavir; UMF HCL, Umifenovir hydrochloride; MOX, Moxifloxacin; LOP, Lopinavir; RIB, Ribavirin; DEX, dexamethasone; MRI, Magnetic resonance imaging; AMX, Amoxicillin; BDQ, Bedaquiline; LNZ, Linezolid; CFZ, Clofazimine; FQ, F?uoroquinolone; CS, Cycloserine; FAV, Favipiravir; TCZ, Tocilizumab; MDS-RAEB-1, MDS with FAB refractory anemia excess blasts-1 subtype; NTEP, National Tuberculosis Elimination Program; MP, Methylprednisolone; CTZ, Ceftazidime; OSE, Oseltamivir; CA, Clavulanic acid; ACs, Anti-coagulant; PYR, Pyrazinamide; CNS, Central nervous system; PNS, Peripheral nervous system; MDS/RAEB, Myelodysplastic syndrome/Refractory anemia excess blasts; ARDS, Acute respiratory distress syndrome; COPD, Chronic obstructive pulmonary disease*.

Regarding TB, the main diagnostic methods in eligible studies were chest X-ray (CXR), smear, and GeneXpert. CT scan served as a diagnostic tool in 21 (67.74%) COVID-19 articles. In addition to CT scan, RT-PCR for COVID-19 was present in 25 (80.64%) papers as inclusion criteria.

A summary of the case report and case series findings is shown in Table 2. Of the total patients, 114 (78.082%) and 32 (21.917%) were men and women, respectively. The most frequent comorbidities in the included studies were smoking (*n* = 22; 15.068%), diabetes (*n* = 21; 14.383%), hypertension (*n* = 13; 8.904%), and alcoholism (*n* = 11; 7.534%). CT images commonly showed ground-glass opacification (GGO) patterns (*n* = 44; 30.136%). The most common treatment drugs for COVID-19 were hydroxychloroquine (*n* = 55; 63.953%), lopinavir/ritonavir (*n* = 34; 39.534%), and azithromycin (*n* = 23; 26.744%).

**Table 2:** Summary of the case report and case series findings.

	**Variables**	**Number**	n/N^*^	**%**
Other Comorbidities	Smoking	22	22/146	15.07%
	Diabetes	21	21/146	14.38%
	Low cellular immunity	1	1/146	0.68%
	Aspergillus fumigatus infection	1	1/146	0.68%
	MDR-TB	4	4/146	2.74%
	Hypertension	13	13/146	8.90%
	Seizure disorder	2	2/146	1.37%
	Chronic liver disease	1	1/146	0.68%
	Schistosomiasis	1	1/146	0.68%
	Without BCG vaccination	1	1/146	0.68%
	HIV	10	10/146	6.85%
	Anemia	3	3/146	2.05%
	Immune-suppression	9	9/146	6.16%
	Glioma	1	1/146	0.68%
	Group A streptococcal	1	1/146	0.68%
	pharyngitis			
	Hepatitis B	1	1/146	0.68%
	Drug abuse	5	5/146	3.42%
	MDS/RAEB myeloid	1	1/146	0.68%
	neoplastic disease			
	Kidney disease	6	6/146	4.11%
	Klebsiella pneumoniae	1	1/146	0.68%
	infection			
	Burning	1	1/146	0.68%
	Alcoholism	11	11/146	7.53%
	Cirrhosis	1	1/146	0.68%
	Pregnancy	6	6/146	4.11%
	XDR-TB	1	1/146	0.68%
	ARDS	5	5/146	3.42%
	Preeclampsia	1	1/146	0.68%
	Parkinson's	1	1/146	0.68%
	Asthma	1	1/146	0.68%
	Hemoptysis	1	1/146	0.68%
	Hypoxemia	2	2/146	1.37%
	Bacterial infection	3	3/146	2.05%
	COPD/asthma	8	8/146	5.48%
	Liver disease	7	7/146	4.79%
	Heart disease	6	6/146	4.11%
	Lung disease	9	9/146	6.16%
Clinical manifestations	Fever	115	115/146	78.77%
	Cough	93	93/146	63.70%
	Dyspnea	27	27/146	18.49%
	Chest pain	3	3/146	2.05%
	Weight loss	12	12/146	8.22%
	Ground glass opacities	44	44/146	30.14%
	Fatigue	6	6/146	4.11%
	Wheeze	3	3/146	2.05%
	Chills	3	3/146	2.05%
	Night sweats	4	4/146	2.74%
	Tachypnea	3	3/146	2.05%
	Tachycardia	3	3/146	2.05%
	Myalgia	5	5/146	3.42%
	Sore throat	2	2/146	1.37%
	Chest pain	3	3/146	2.05%
	Emphysema	2	2/146	1.37%
	Anxious	1	1/146	0.68%
	Respiratory distress	33	33/146	22.60%
	Hypoxia	2	2/146	1.37%
	Sepsis	1	1/146	0.68%
	Hemoptysis	3	3/146	2.05%
	Headache	6	6/146	4.11%
	Increased expectoration	1	1/146	0.68%
	Asthenia	1	1/146	0.68%
	Poor appetite	4	4/146	2.74%
	Anosmia	1	1/146	0.68%
	Dysgeusia	1	1/146	0.68%
	Nocturnal diaphoresis	1	1/146	0.68%
	Dizziness	2	2/146	1.37%
	Vomiting	1	1/146	0.68%
	Brain inflammation	1	1/146	0.68%
	Abdominal pain	2	2/146	1.37%
	Abdominal distension	1	1/146	0.68%
	Orthopnea	1	1/146	0.68%
	Shivering	1	1/146	0.68%
	Fair mental status	1	1/146	0.68%
	Poor sleep	1	1/146	0.68%
	Muscle pain	1	1/146	0.68%
	Body aches	1	1/146	0.68%
	Diarrhea	1	1/146	0.68%
	Chest tightness	2	2/146	1.37%
Outcomes	Discharge	100	100/115	86.96%
	Death	15	15/115	13.04%
TB disease	Pulmonary	91	91/116	78.45%
	Disseminated	14	14/116	12.07%
	Extra-pulmonary	8	8/116	6.90%
	CNS	2		
	Pleural	2		
	Cervical	1		
	PNS	1		
	Abdominal	1		
COVID-19 treatment	HCQ	55	55/86	63.95%
	Lop/Rit	34	34/86	39.53%
	AZT	23	23/86	26.74%
	Oxygen therapy	17	17/86	19.77%
	Arbidol	6	Jun-86	6.98%
	Corticosteroid	4	Apr-86	4.65%
	MOX	3	Mar-86	3.49%
	Acetyl cysteine	3	Mar-86	3.49%
	MP	3	Mar-86	3.49%
	CTX	3	Mar-86	3.49%
	IFN-α	2	Feb-86	2.32%
	Prednisone	2	Feb-86	2.32%
	DEX	2	Feb-86	2.32%
	ACs	2	Feb-86	2.32%
	Plasma therapy	2	Feb-86	2.32%
	Vit C	2	Feb-86	2.32%
	LNZ	2	Feb-86	2.32%
	Thymosin	1	Jan-86	1.16%
	Piperacillin/tazobactam 1	1	Jan-86	1.16%
	Clarithromycin	1	Jan-86	1.16%
	Paracetamol	1	Jan-86	1.16%
	AMX/CA	1	Jan-86	1.16%
	Zinc Sulfate	1	Jan-86	1.16%
	OSE	1	Jan-86	1.16%
	TCZ	1	Jan-86	1.16%
	FAV	1	Jan-86	1.16%
	CS	1	Jan-86	1.16%
	AMX	1	Jan-86	1.16%
	RIB	1	Jan-86	1.16%
	Dolutegravi	1	Jan-86	1.16%
	Lamivudine	1	Jan-86	1.16%
	Tenofovir	1	Jan-86	1.16%
	LEV	1	Jan-86	1.16%
	Co-AMX	1	Jan-86	1.16%
	IG	1	Jan-86	1.16%
	UMF HCL	1	Jan-86	1.16%
Gender	Male	114	114/146	78.08%
	Female	32	32/146	21.92%

* *n, number of patients with any variables; N, the total number of patients with COVID-19*.

Extrapulmonary TB (EPTB) and disseminated form were reported in eight and 14 (12.068 vs. 6.896%) TB cases. Concerning viral and bacterial infectious diseases, 10 (6.849%), one (0.684%), and five (3.424%) patients had HIV, HBV, and bacterial infection, respectively. In addition, MDR and XDR *M. tuberculosis* strains were identified in four and one patient, respectively. The most frequent clinical complications were fever (78.767%), cough (63.698%), GGO (30.136%), dyspnea (18.493%), and respiratory distress (22.602%).

The present systematic review reported that the mortality rate in patients with TB/COVID-19 coinfection was 13.04% and the rate of discharged patients was 86.96%.

## Discussion

The novel coronavirus has declared as a public health emergency of international concern on January 31, 2020. The virus has had irreversible effects on the health and economy of the world and has placed limitations on society (48). TB is the most important cause of death through a single infectious agent (49, 50). Both *M. tuberculosis* and SARS-CoV-2 are transmitted through the respiratory tract and often affect the lungs. Thus, there are concerns about the effect of COVID-19 on the clinical course of TB and its outcome (49). In this study, we described the different dimensions of concurrent COVID-19 and TB infection.

The most common clinical manifestations in TB patients include coughing up mucus or blood, chronic cough (>2 months), chest pain, loss of appetite, weight loss, and chills (21). In contrast, the most clinical manifestations of COVID-19 include fever, cough, dyspnea, muscle ache, confusion, and headache, respectively (51). The findings of the present study introduce different clinical manifestations for coinfection with TB and COVID-19. The highest incidence of clinical manifestations belonged to fever, cough, ground-glass opacities, respiratory distress, dyspnea, and weight loss with 78.767, 63.698, 30.136, 22.602, 18.493, and 8.219%, respectively. COVID-19 has rapid clinical manifestation, but TB is time-consuming, and the onset of symptoms takes longer. This feature can help differentiate between the two diseases.

Our study has demonstrated that the most common comorbidities were smoking, diabetes, hypertension, alcohol consumption, and HIV. Stochino et al. have shown that old age, diabetes, and respiratory diseases are the main factors increasing the mortality in patients with TB/COVID-19 coinfection (49). In addition, it has proven that respiratory illnesses can cause lung dysfunction and reduce resistance to the virus. Thus, TB patients are more likely to develop severe COVID-19 (19).

Tuberculosis and COVID-19 have similarities in symptoms and chest radiographs that can lead to misdiagnosis (31). COVID-19 aggravates or obscures latent TB, leading to increased TB mortality (18). Motta et al. did not consider TB a leading cause of death and reported higher mortality rates in the elderly with coinfection than the others (52). In dead patients, COVID-19 infection was during the primary phases of the epidemic, indicating the importance of careful implementation of infection-control interventions for inpatients and hospital staff (52). Although the signs and symptoms of the two diseases are similar, the treatment and clinical course are different (31). Anti-TB therapies are not effective against COVID-19 because COVID-19 occurs during the use of these therapies (53). According to this study, most treatment of COVID-19 includes hydroxychloroquine (63.95%), lopinavir/ritonavir (39.53%), azithromycin (26.74%), and oxygen therapy (19.7%). Stochino et al. have observed no drug interactions between anti-TB drugs and hydroxychloroquine (49). On March 28, 2020, hydroxychloroquine received an emergency use permit for use in patients with COVID-19. However, clinical trials have shown that the drug has no particular advantage in treating COVID-19, so the FDA revoked the drug's emergency license (54).

Mortality rate was reported among patients coinfected with TB and COVID-19 (18, 31). A systematic review conducted by Nasiri et al. in 2020 reported that the pooled mortality rate from COVID-19 was 6.6% (55). The present review includes case reports and case series around the world. The mortality rate in patients with concurrent infection of TB and COVID-19 in this study is 13.04%, and the rate of discharging from the hospital is 86.96%. The difference in mortality rates in the two studies mentioned above indicates that the coinfection of TB and COVID-19 increases mortality.

Various viruses such as influenza, measles, and HIV cause the malfunction of macrophages that inhibit the growth of *M. tuberculosis* (56, 57). TypeI IFN signaling pathway plays a key role in increasing the growth of *M. tuberculosis* among patients coinfected with respiratory viruses and *M. tuberculosis* (58). Intracellular growth and response of macrophages to IFN-γ increases with increasing typeI interferon (59). Interferon-gamma is highly present in the early stages of COVID-19 (60). Therefore, COVID-19 is a risk factor for TB (31). COVID-19 weakens the immune system in various ways: (1) Destroying lymphocytes (2) destroying B cells (3) damage to T-cell (4) damage to NK cell (61). Lymphocyte depletion is one of the common causes of coinfection (62). The immune system, especially CD4 T cell-mediated immunity, is exposed to TB. In addition, both *M. tuberculosis* and *SARS-CoV-2* can interfere with the immune system. It leads to immunodeficiency and lymphopenia with a decrease in CD4+ T cells. (31). Lymphopenia is a good indicator of the severity of the disease. Defected immune systems worsen in patients with COVID-19. On the other hand, TB causes respiratory failure in patients with COVID-19 due to interference with the cellular immune system and lymphopenia (31).

In 2020, Nigeria reported a decreasing number of diagnoses and possible cases of TB compared with 2019, which could be due to the prevalence of COVID-19 and restrictions on the assessment of diagnosis and uninterrupted treatment of TB (53). Lai et al. in Taiwan declared that the prevalence of TB decreased in the first 20 weeks of 2020 (63). Failure to diagnose TB promptly not only delays the treatment of patients but also infects others. On the other hand, late treatment also increases the severity of the disease and consequently increases the mortality rate. Since the global health system today focuses on COVID-19, the TB control program has received less attention. This has led to delays in the “End TB Strategy” (21). With proper care, the effect of COVID-19 on active TB can be controlled clinically (49).

SARS-CoV-2/bacteria coinfections can cause problems in the diagnosis, treatment, and prognosis of COVID-19 and increase the symptoms of the disease and mortality rate (11). According to the results of a systematic review by Gao et al., the prevalence of TB among COVID-19 patients was 0.37 to 4.47% (19). To prevent coinfection of TB and COVID-19 during the epidemic, physicians should be aware of the following: (1) Pneumonia associated with COVID-19 (2) Methods of distinguishing TB and COVID-19 from each other (3) The differences between TB and COVID-19 (31).

The COVID-19 epidemic has led to a decline in immunization services and increase in deaths from vaccine-preventable diseases (64). Among the vaccines proposed against COVID-19, the BCG vaccine has found a special place to prevent COVID-19 in the scientific community. In addition, the BCG has nonspecific effects on the immune system that lead to protective immunity from other infections. The BCG vaccine may reduce viremia after exposure to SARS-CoV-2 and decrease the severity of COVID-19 (65). People with active and latent TB should avoid COVID-19. Therefore, appropriate vaccination should be provided for them (21).

There are several limitations to this review. First, only case report/case series studies were included in this review, and the observational studies were excluded because of their absence or insufficient data. Second, case reports and case series are more likely to be biased than other studies. Third, case studies and case series are descriptive and describe the patient's signs and symptoms. The prevalence and percentage of coinfection in them have not been studied. For this reason, it was not possible to perform metaanalysis calculations in this review. Therefore, the prevalence of TB among COVID-19 patients and vice versa has not been calculated. Fourth, one of the problems with the SARS-CoV-2 virus is the existence of new variants (66). In recent months, the appearance of a new variant of this virus called the SARS-CoV-2 Delta variant (B.1.617.2) has spread in different parts of the world. This variant was first found in India and, its main feature is its high ability to transfer and escape from the immune system (67). One of the challenges ahead in curbing the spread of *M. tuberculosis* during the COVID-19 epidemic is the new variants of the virus. However, the extent to which new variants may play a role in the prevalence of coinfection has not yet been determined and requires further and more extensive studies.

## Conclusions

There have been many reported cases of viral, fungal, and bacterial infections associated with COVID-19. In this study, we studied the association between TB and COVID-19. We discussed clinical characteristics, diagnosis, treatment, and mortality rate of TB/COVID-19 coinfectetd patients. Patients with TB are more likely to get COVID-19 infection. Testing TB patients for COVID-19 and vice versa is a controversial topic in the scientific community. COVID-19 and TB, although slightly different in how they are transmitted, have similar clinical features and manifestations such as fever, shortness of breath, and cough. However, these symptoms are also slightly different from each other. A noteworthy point in distinguishing these two diseases from each other is the time of onset of clinical symptoms, which is rapid in patients with COVID-19 but time-consuming in people with TB. Since the symptoms of both the diseases are similar, it is difficult to diagnose them, and sometimes the diagnosis of TB occurs later, which causes the progression and severity of the disease. Both diseases have similar risk factors, such as old age, diabetes, immunodeficiency, HIV, and COPD. Several reasons show that TB can facilitate COVID-19 establishment. One of these factors is related to the damage caused by TB in the lungs that predispose a person. The second reason is the immune system of a patient with TB that can increase the susceptibility to COVID-19. Finally, a regular program is recommended to detect TB during the outbreak of COVID-19 and follow it up continuously to prevent the occurrence of these two diseases simultaneously.

## Author Contributions

MH and SK designed the study and revised the manuscript. MH, MK, AN, NM, PM, FT, and SG performed the search, acquisition of data, and wrote the first draft of the article. MH and NM analyzed and interpreted the data. All authors contributed to the article and approved the submitted version.

## Conflict of Interest

The authors declare that the research was conducted in the absence of any commercial or financial relationships that could be construed as a potential conflict of interest.

## Publisher's Note

All claims expressed in this article are solely those of the authors and do not necessarily represent those of their affiliated organizations, or those of the publisher, the editors and the reviewers. Any product that may be evaluated in this article, or claim that may be made by its manufacturer, is not guaranteed or endorsed by the publisher.
